# Diagonistic Apraxia: A Unique Case of Corpus Callosal Disconnection Syndrome and Neuromyelitis Optica Spectrum Disorder

**DOI:** 10.3389/fneur.2018.00653

**Published:** 2018-08-10

**Authors:** Hidenobu Shozawa, Akinori Futamura, Yu Saito, Motoyasu Honma, Mitsuru Kawamura, Michael W. Miller, Kenjiro Ono

**Affiliations:** ^1^Division of Neurology, Department of Internal Medicine, School of Medicine, Showa University, Tokyo, Japan; ^2^Neurology, Okusawa Hospital and Clinic, Tokyo, Japan; ^3^Postgraduate School of Medicine, University of Tokyo, Tokyo, Japan

**Keywords:** neuromyelitis optica spectrum disorder, corpus callosum disconnection syndrome, diagonistic apraxia, anti-aquaporin-4 antibody, corpus callosum fibers

## Abstract

Diagonistic apraxia is a corpus callosal disconnection syndrome. Callosal lesions in Neuromyelitis optica spectrum disorder (NMOSD) have been reported, but callosal disconnection syndrome are rare. A 48-year-old woman was treated for fever and a cough before hospitalization. Her fever abated immediately, but she had balance problems in walking and standing. She also had slurred speech. On neurological examination, she had diagonistic apraxia. Her left hand moved in an uncoordinated way when she moved her right hand: changing her clothes for example or using a knife and fork. She had to instruct her left hand to stop. She had dysarthria and her gait was wide-based. She also had many callosal disconnection syndrome symptoms such as alexia of left visual field, left ear extinction, crossed optic ataxia. Using FLAIR and DWI MRI, a mixture of low and high signals, a so-called “marbled pattern,” was seen in the corpus callosum. Since the patient was positive for anti-aquaporin-4 antibody, she was diagnosed with NMOSD. After two courses of steroid pulse therapy, the symptoms improved. Here we report diagonistic apraxia and other symptoms of callosal disconnection syndrome in anti-AQP4-positive NMOSD.

## Background

The unique aspect of this case was diagonistic apraxia, in which the patient's hands were uncoordinated, and the left hand had to be verbally instructed to follow the patient's will. Liepmann and Mass first reported the symptoms in 1907 (1). Diagonistic apraxia is a corpus callosal disconnection syndrome. Different symptoms are reported for lesions in different segments on the corpus callosum. Lesions in the posterior truncus are related to diagonistic apraxia, crossed tactile anomia and tactile disorientation. Lesions in the splenium are related to alexia of left visual field, and lesions involving both the splenium and posterior truncus are related to auditory extinction of the left ear and crossed optic ataxia (2). Impaired prosody in speech is associated with lesions predominantly involving the anterior corpus callosum (3). Neuromyelitis optica spectrum disorder (NMOSD) is an inflammatory disease caused by necrotic demyelination, mainly of the optic nerve and spinal cord and anti-aquaporin-4 antibody is an NMO-specific autoantibody. Callosal lesions in NMOSD have been reported, but callosal disconnection syndrome is rare (4).

## Case reports

A forty-eight year-old, right-handed female was admitted for gait disturbance and unusual movement of the left hand. She was treated for fever and cough before hospitalization. While her fever abated immediately, she had balance problems in walking and standing. Her past medical history was uneventful and there was no family history of optic neuritis, myelitis or NMOSD. She was alert and had no abnormal physical findings. On neurological examination, there was no weakness in muscles and no sensory disturbance, but she had uncoordinated movement in her both lower limbs in performing heel-knee test, and her gait was wide-based, and could not perform a tandem gait test. Neuropsychological examination revealed signs of the following corpus callosal disconnection syndromes: diagonistic apraxia, tactile anomia in the left hand, crossed tactile disorientation, crossed optic ataxia, alexia of the left visual field, auditory extinction of the left ear.

With diagonistic apraxia, she experienced bizarre movements in her left hand while changing her clothes and having meals. When taking a meal, her left hand involuntarily grabbed her rice bowl, while her right hand was selecting and picking food from a dish with chopsticks. The errant hand came under control when reprimanded verbally.

In laboratory testing, erythrocyte sedimentation rate was elevated 30 mm/1 h and 48 mm/2 h. Anti-aquaporin 4 (AQP4), Anti-Sjögren's syndrome-related antigen A (anti-SS-A), anti-SS-B, and anti-Mycoplasma pneumoniae-IgM antibodies were positive. Cerebrospinal fluid findings were lymphocytosis (9/μL), elevated protein content of 52 mg/dL, elevated myelin basic protein level of 1221.5 pg/mL (normal <102 pg/ml) and elevated IgG index of 0.9 (normal <0.8). The oligoclonal bands were negative. Her chest X-ray and CT examinations were normal.

**Figure 1:**
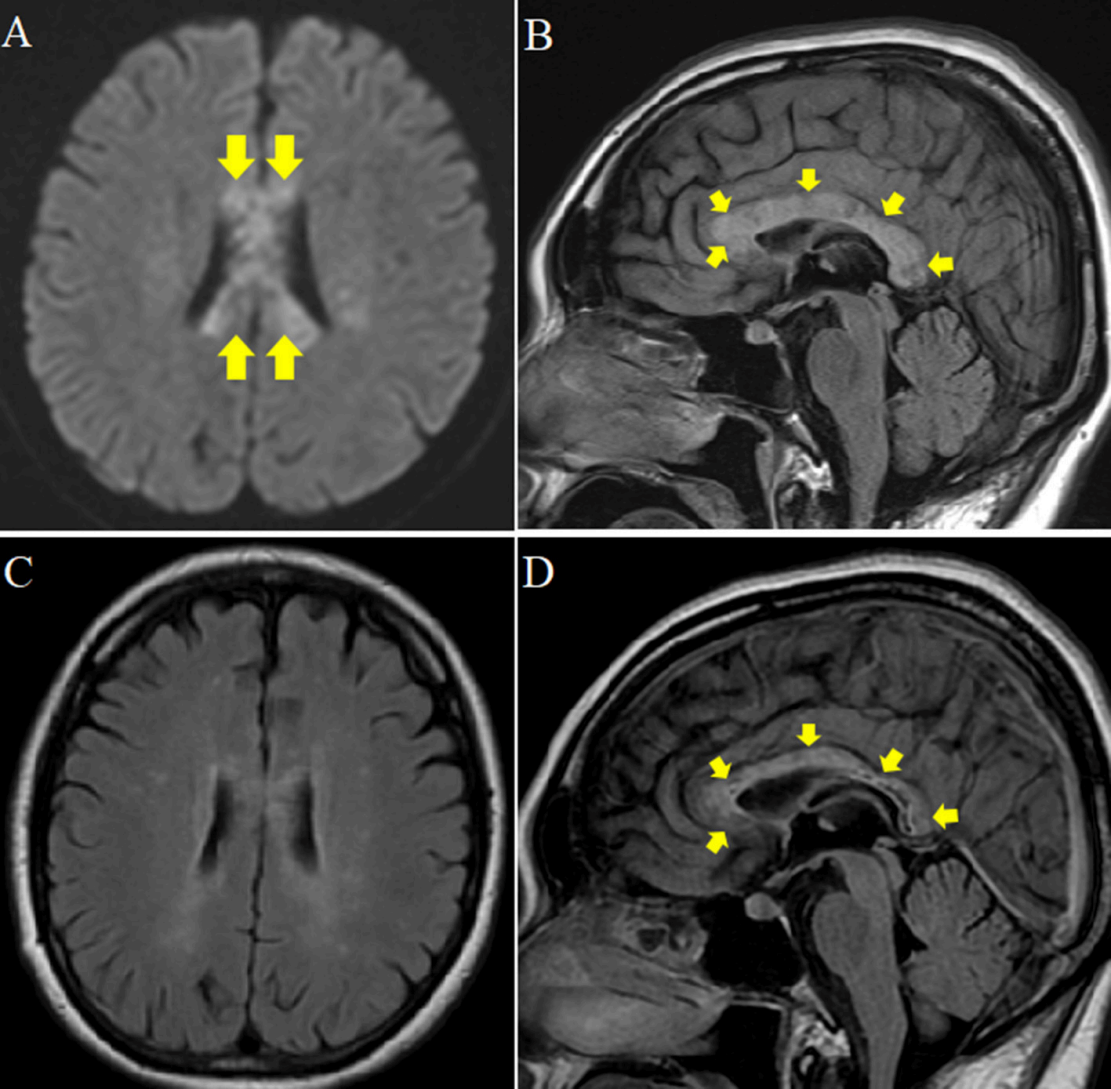
Brain MRI at the beginning of hospitalization and 1 year later. **(A,B)** Plain MRI at the beginning of hospitalization. **(A)** Diffusion weighted MRI axial view showing patchy high signal intensity in the corpus callosum (arrow). **(B)** Fluid Attenuated Inversion Recovery (FLAIR) midline sagittal view showing edematous and irregular intensity and lower intensity at the core (arrow). **(C,D)** Plain MRI 1 year after hospitalization. **(C)** FLAIR axial view showing improved patchy high intensity in the corpus callosum. **(D)** FLAIR midline sagittal view shows regression of edema and ill-defined margin at the rim of splenial lesions. However, irregular signal, and low intensity at the core remained (arrow).

Brain MRI showed high-signal intensities of the entire corpus callosum from genu to splenium (Figures 1A,B). The lesions on the corpus callosum were a mixture of patchy low and high-intensity signals. She had prodromal infection, elevation of cells and myelin basic protein in cerebrospinal fluid, and lesions in the corpus callosum on brain MRI. Multiple sclerosis, NMOSD, acute disseminated encephalomyelitis, clinically mild encephalitis/encephalopathy with a reversible splenial lesion due to mycoplasma infection or vasculitis were considered as differential diagnoses. Because Anti-AQP4, anti-SS-A, and anti-SS-B antigens were positive and salivary gland biopsy revealed inflammatory cell infiltration with lymphocytes and plasma cells, the patient was diagnosed with anti-AQP4-seropositive NMOSD with SS (5).

After intravenous steroid pulse therapy (1,000 mg of methylprednisolone for 3 days) was performed twice and the following symptoms improved; ataxia of the legs, unsteadiness in standing and walking, callosal disconnection syndromes such as left-hand diagonistic apraxia, left hand tactile anomia, crossed tactile disorientation, crossed optic ataxia, and left-visual field alexia. However, the patient's corpus callosum lesion was still edematous on MRI, and auditory extinction of left ear remained. Intravenous immunoglobulin (IVIg) was begun on day 21 for 5 days but changed little. She was discharged on day 40 with subsequent treatment of tapered prednisolone at 40 mg/day. Azathioprine 100 mg was also added. One year later the left-field alexia disappeared. A mottled signal inside the corpus callosum also remained (Figures 1C,D).

## Discussion

Diagonistic apraxia is a unique disconnection syndrome, in which a patient's hands are uncoordinated, and the left hand has to be verbally instructed to follow a patient's will. Liepmann and Mass first reported the symptoms in 1907 (1). The patient had agraphia and apraxia on the left side without language comprehension impairment. In diagonistic apraxia, uncooperative movements are triggered in the left hand on moving the right. During right hand movement, the left interrupts the right repeatedly to obstruct the action. The genu and anterior trunk of the corpus callosum are thought to be involved (6).

The left hand moved against the patient's will until she verbally commanded to stop in this patient. The left hemisphere is conscious and dominates verbal behavior. The right hemisphere comprehends simple questions and responds effectively with non-verbal responses. The left hemisphere integrates thoughts to mediate consciousness closely linked with linguistic function, which Gazzaniga called the “integrator system” (7). The disconnection with the left hemisphere the left hand moved against the one's will.

In the present case, the entire corpus callosum was affected and linked to various disconnection syndromes. Lesions of the entire corpus callosum are rare. There are also no previous reports of disconnection syndrome in NMOSD. This is, we believe, the first case revealing disconnection syndrome in NMOSD with lesions of the entire corpus callosum.

Nakamura reported callosal lesions in four acute NMOSD patients out of 22 (8). On FLAIR imaging, these were multiple, edematous, and large, measuring at least 10 mm, and presenting a “marbled pattern” with higher intensity at the rim and lower intensity at the core. Concurrent splenial lesions characteristic of NMOSD were also identified in this patient.

The density of corpus callosal fibers and marrow vary across the structure (9). The commissural fibers are thin at the genu, thick in the truncus, and of mixed size in the splenium (10, 11). The vulnerability of the splenium is attributable to the fact that cellular fluid is easily perturbed, and impaired water excretion due to loss of AQP4 is greater in splenial lesions (12). Auditory extinction of left ear persisted because edema lesions were splenial as opposed to rostral and affected the genu and truncus.

## Concluding remarks

Callosal disconnection syndrome can develop as a type of brain disorder related to anti-AQP4-antibody-positive NMOSD.

## Ethics statement

The Ethics Committee of Showa University School of Medicine approved this study (No.287), and it was performed according to the Declaration of Helsinki. Written patient consent was obtained for publication of this case report.

## Author contributions

HS, AF, YS, and, MK examined the patient. HS, AF, YS, MK, MM, and KO discussed the case. HS, AF, and KO wrote the manuscript, and MH, MK, MM, and KO reviewed it. All authors approved the manuscript.

### Conflict of interest statement

The authors declare that the research was conducted in the absence of any commercial or financial relationships that could be construed as a potential conflict of interest.
